# A case-control study of early-life residential exposure to tetrachloroethylene and risks of childhood cancer and birth defects

**DOI:** 10.1016/j.envint.2025.109600

**Published:** 2025-06-10

**Authors:** Jongeun Rhee, Adrian M Michalski, Margaret Gates Kuliszewski, Jamie Musco, Catherine T Adler, Shuai Xie, Melissa C Friesen, Tabassum Insaf, Mark P. Purdue

**Affiliations:** a Occupational and Environmental Epidemiology Branch, Division of Cancer Epidemiology and Genetics, National Cancer Institute, Rockville, MD, USA; b Bureau of Environmental & Occupational Epidemiology, New York State Department of Health, Albany, NY, USA; c Bureau of Cancer Epidemiology, New York State Department of Health, Albany, NY, USA; d Birth Defects Registry, New York State Department of Health, Albany, NY, USA; e Department of Epidemiology & Biostatistics, University at Albany, Albany, NY, USA

**Keywords:** Tetrachloroethylene, Dry-cleaning facility, Childhood cancer, Birth defects, Aortic valve stenosis

## Abstract

**Background:**

Residential buildings with a co-located dry-cleaning facility (CL-DC) can have substantially higher indoor tetrachloroethylene concentrations than buildings without a CL-DC. We conducted a case-control study to investigate associations between early-life indoor tetrachloroethylene exposure from CL-DCs and risks of childhood cancers (overall, acute lymphoblastic leukemia) and birth defects.

**Methods:**

We linked records between the New York City (NYC) Bureau of Vital Statistics and the New York State Cancer Registry and Birth Defects Registry to identify cases of childhood cancers (n = 5,334) and birth defects (n = 171,553) diagnosed among children born in NYC between 1988 and 2016, and controls without these conditions (n = 596,599). We identified CL-DC exposure by mapping addresses from birth certificates and DC permits involving tetrachloroethylene use to building footprints, and modeled tetrachloroethylene concentrations using measurement data from a survey of NYC CL-DCs. Using unconditional logistic regression, we computed odds ratios (ORs) and 95 % confidence intervals (CIs) relating study endpoints to CL-DC exposure.

**Findings:**

Living in a building with a CL-DC at birth was associated with aortic valve stenosis (AVS; OR = 3.1, 95 % CI = 1.6, 5.9), with an exposure–response effect for predicted tetrachloroethylene concentration (≤44 μg/m^3^: OR = 2.7, 95 % CI = 1.0, 7.4; >44 μg/m^3^: OR = 3.9, 95 % CI = 1.6, 9.5) and stronger associations for children whose mother was non-White, less than college-educated, or lived at birth in majority non-White or poorer neighborhoods. We observed null findings for other endpoints.

**Conclusions:**

In this first-ever case-control study, early-life tetrachloroethylene exposure from CL-DCs in residential buildings was associated with increased AVS risk. These findings warrant further investigation.

## 1. Introduction

Tetrachloroethylene (“perc”) is a chlorinated solvent widely used in the dry-cleaning industry since the 1960 s. (US Environmental Protection Agency, 2020; US Environmental Protection Agency, 2017) The U. S. Environmental Protection Agency estimated that the percentage of U. S. dry-cleaning facilities using perc as a solvent was 83 % in 1991, and 60 % by 2017. (US Environmental Protection Agency, 2017) There has been longstanding concern over the health effects of perc. (US Environmental Protection Agency, 2020; Guyton et al., 2014) In 2012, the International Agency for Research on Cancer classified perc as a probable human carcinogen (2A) on the basis of sufficient evidence in experimental animals and limited human evidence. (International Agency for Research on Cancer, 2014) Existing epidemiologic evidence for cancer has come mainly from occupational studies of dry-cleaning industry workers and related adult-onset malignancies such as hematologic cancers and cancers of the bladder and kidney, (International Agency for Research on Cancer, 2014; Blair et al., 2003) although there is also suggestive epidemiologic evidence of an elevated risk of childhood leukemia. (Sung et al., 2008; Reynolds et al., 2003) There is also evidence from several epidemiologic studies suggesting associations with reproductive and developmental toxicity among perc-exposed female workers and women living in areas with perc-contaminated water. (US Environmental Protection Agency, 2020; Aschengrau et al., 2018; Ruckart et al., 2014; Aschengrau et al., 2009; Bove et al., 1995; Doyle et al., 1997).

Perc is primarily released into the environment from commercial and industrial sources. (Nijhuis et al., 2010) Environmental perc exposure is of particular concern for residents in buildings with co-located dry-cleaning facilities (CL-DCs), as fugitive perc emissions can contaminate indoor air throughout the buildings. (Schreiber et al., 1993; McDermott et al., 2005) Investigations of perc emissions in residential buildings with CL-DCs have been conducted in New York State (NYS), with surveys conducted between 1991 and 1997 by the New York State Department of Health (NYSDOH) reporting geometric mean concentrations of perc in air samples from CL-DCs ranging as high as 55,000 μg/m^3^. (Schreiber et al., 1993; McDermott et al., 2005; Wallace et al., 1995) These findings led to the implementation of new regulations in NYS in 1997 aimed at reducing fugitive perc emissions within CL-DCs. (New York State Department of Environmental Conservation) A New York City (NYC) survey conducted between 2001 and 2003 demonstrated a reduction in indoor perc concentrations in residential buildings with CL-DCs following these regulations, although the concentrations remained over 10-fold higher than in buildings without a CL-DC. (McDermott et al., 2005) In particular, perc levels were over 25 times higher in buildings with CL-DCs located in majority non-White neighborhoods and 85 times higher for buildings in low-income neighborhoods compared to buildings without CL-DCs. (McDermott et al., 2005) In 2005, the U.S. Environmental Protection Agency introduced a rule calling for the phase-out of perc use in residential CL-DCs by 2020; (Environmental Protection Agency, 2005) notably, the use of perc in a residential building has not been restricted in most other countries.

To our knowledge, no studies to date have investigated disease risks associated with residential perc exposure from CL-DCs other than for neurobehavioural and visual contrast sensitivity testing. (Schreiber et al., 2002; Altmann et al., 1995) Children may be particularly vulnerable to residential perc exposures given the timing of exposures *in utero* and in early life, when major developmental processes are ongoing. To address the previously unexplored relationship between early-life residential exposure to perc from CL-DCs and risks of birth defects and childhood cancers, we conducted a record linkage-based case-control study within NYC.

## 2. Methods

### 2.1. Study Design

The source population for this case-control study included children born in NYC (as determined by the mother’s address on the birth certificate) between January 1, 1988 and December 31, 2016 with a birth record at the Office of Vital Statistics of the NYC Department of Health and Mental Hygiene (NYCDOHMH). To facilitate linkages to residential buildings, the NYCDOHMH identified for each mother’s residential address on the birth records the corresponding NYC Building Identification Number (BIN), a unique identifier assigned to NYC buildings by the Geographic Systems Section of the NYC Department of City Planning, using Geosupport System software. After excluding birth records with a missing BIN over the study period, 1,058,501 eligible birth records were identified.

We identified the case group of children diagnosed with cancer at age 19 or younger between 1988 and 2016 (n = 5,334) from among the eligible birth records through linkage of the NYC birth records (NYC Health) to the NYS Cancer Registry (New York State Department of Health) through probabilistic matching based on the child’s full name, date of birth and sex. Uncertain matches were manually reviewed using additional available information including parental name, address and phone number at diagnosis, and residential history. The NYS Birth Defects Registry (New York State Department of Health), a passive registry, is routinely linked to NYCDOHMH birth records through probabilistic matching based on the child’s date of birth, child’s last name, mother’s date of birth, child’s medical record number and ZIP code of the birth record; we identified 171,553 cases with one or more major birth defects from among the eligible birth records. We excluded cases with birth defects of known etiology (e.g. fetal alcohol syndrome, diagnoses and syndromes related to maternal infections, Down syndrome, trisomy 13, trisomy 18). Additionally, birth defects with known ascertainment issues given the passive nature of the birth defect registry (e.g. atrial septal defect, ventricular septal defect, patent ductus arteriosus, congenital hip dislocation, and amniotic bands) were excluded from individual level analysis. A detailed description of the birth defect registry and the ascertainment methods used can be found in the Supplementary Method 1. The study originally included a third case group involving fetal deaths identified from NYCDOHMH fetal death certificate records within the study period (n = 284,967); however, this case group was later excluded from analysis due to issues of incomplete or missing information regarding mother’s address on fetal death records and identified inaccuracies in the assignment of BINs to available addresses. We selected a control group from among live births with no records of cancer or birth defect diagnoses, frequency matched to the distribution of year of birth for all case groups combined. We included a number of controls equivalent to the total sum of cases across all three case groups, increased by an additional 20 % (in case of additional exclusions at the exposure assessment step), for a total of 596,599 controls.

### 2.2. Perc exposure assessment

The NYC Department of Environmental Protection (DEP) requires that all dry-cleaning facilities using perc hold a valid certificate of operation, renewable every three years. (NYC Environmental Protection) We used a database of certificates of operation filed with NYC DEP between 1980 and 2020 to identify dry-cleaning facilities using perc over the study period, and converted the addresses of facilities to BINs using Geosupport System V 22.31.

We then linked the BINs of maternal addresses and of dry-cleaning facilities to identify buildings with a co-located perc-using dry-cleaning facility in which a child’s mother lived at the time of birth, as a means of indirectly assessing potential early-life residential exposure to perc. For potential matches we verified whether the year of birth was within the time span between the year that a facility applied for a certificate of operation and its date of expiration or cancellation.

For individuals identified to have lived at birth in a building with a CL-DC, we estimated the level of perc exposure through the development of a model using indoor air concentrations of perc from a survey of NYC residential buildings with CL-DCs conducted between 2001 and 2003, previously published by McDermott et al. (McDermott et al., 2005); a detailed description of the model development methods is provided in the Supplementary Method 2. Briefly, in an analysis of the survey measurement data, we found the total number of floors in a building to be the strongest predictor of elevated indoor perc concentrations from among the measured building and neighborhood characteristics (including the specific floor sampled, whether a building had a prior complaint regarding facility emissions, whether the 2000 Census Block Group population was majority non-Hispanic White, whether ≥ 23.59 % of the 2000 Census Block Group population fell below the poverty threshold). After applying several modeling approaches, we observed that an inverse transformation of total number of floors had the lowest model Aikake Information Criterion (AIC), indicating the most predictive model accounting for parsimony:

$$\mathrm{Predictedgeometricmeanpercconcentrations}\;=\exp(1.00+19.45\times \frac{1}{\;\mathrm{Totalnumberoffloors}})$$

The model-predicted perc concentrations by total number of floors are summarized in Supplementary Table S1 and Supplementary Fig. S1. To apply the model to exposed study participants, we extracted the total number of floors for identified buildings with a co-located dry cleaning facility through linkage of BINs to the MapPLUTO database containing characteristics of tax lots in NYC. (NYC Department of City Planning) We restricted the linkage to tax lots with a single building structure (85 % of all exposed participants).

### 2.3. Statistical analysis

**Fig. 1.**
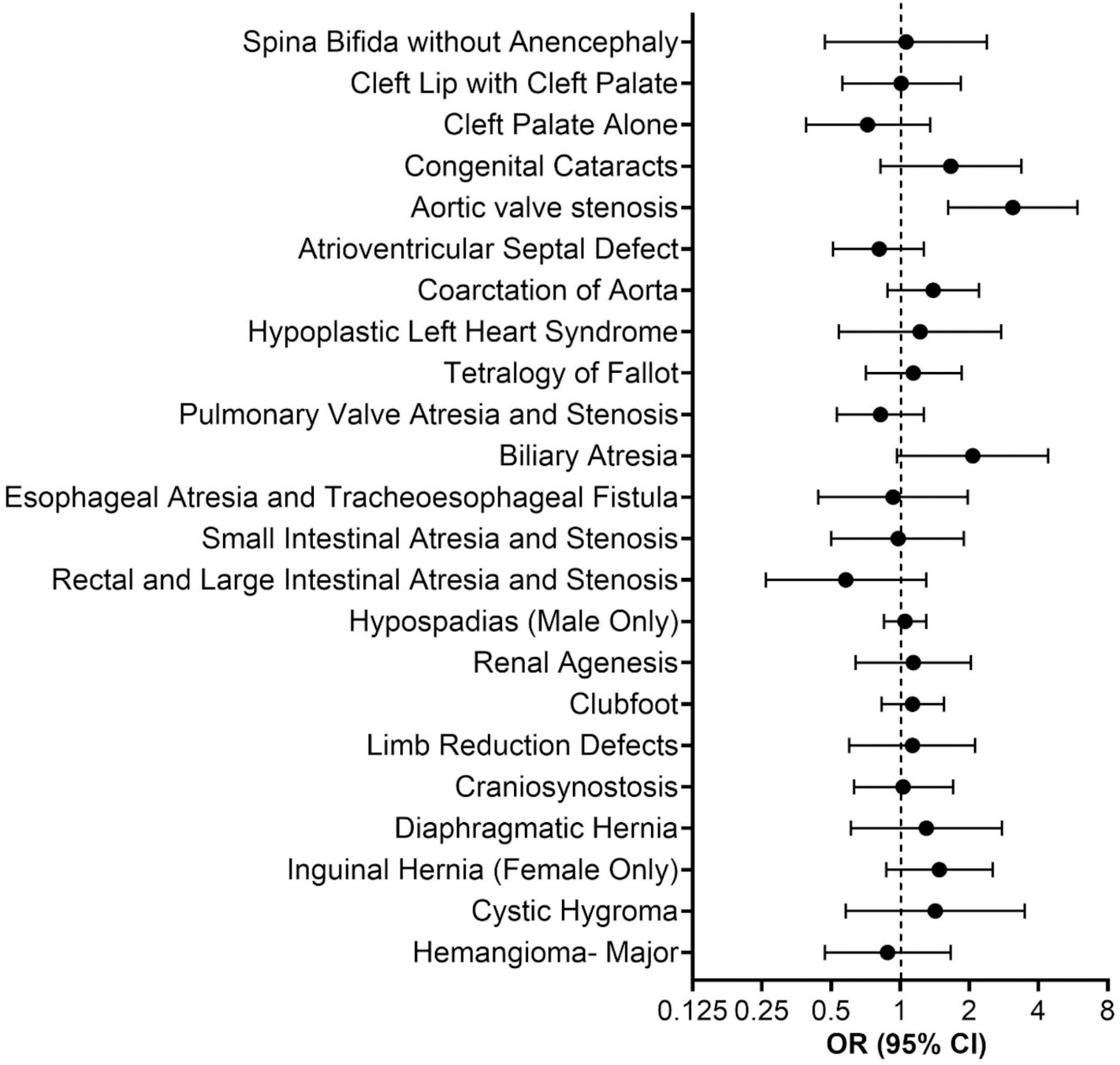
Associations between early-life residence in a building with a co-located dry cleaning facility and selected birth defects (n = 23).

We summarized and compared the distributions of selected participant characteristics by case-control status (among controls, overall childhood cancers, total birth defects) and by exposure to a CL-DC facility (yes, no). For our case-control analyses, we used multivariable unconditional logistic regression to compute odds ratios (ORs) and 95 % confidence intervals (CIs) evaluating associations with exposure for disease endpoints with a minimum of five exposed cases; endpoints satisfying this criterion included overall childhood cancers, acute lymphoblastic leukemia, total birth defects, and the individual birth defects listed in Fig. 1. We evaluated associations with early-life perc exposure both as a dichotomous variable (no exposure, exposed) and across categories of increasing estimated indoor perc concentration (no exposure, ≤44 μg/m^3^, >44 μg/m^3^; 44 μg/m^3^ is the median concentration among controls). To examine linear trends across categories of perc exposure, we used a Wald test by assigning 3 μg/m^3^ (previously reported perc levels within the buildings without CL-DCs^15^), 22 μg/m^3^ (within-category median value), and 44 μg/m^3^ as a continuous variable, respectively. We first fit models adjusting for year of birth (<1995, 1995-<2002, 2002-<2010, 2010 or later), the sole control frequency matching factor. In multivariable models, we further adjusted for borough (Manhattan, Bronx, Brooklyn, Queens, Staten Island), participant sex (male, female), mother’s age (<25, 25–30, 30–34, >35, missing), mother’s race and ethnicity [non-Hispanic White, non-Hispanic Black, Hispanic, other (‘other’ includes non-Hispanic Asian, Native Hawaiian and other Pacific Islander, American Indian and Alaska Native, some other race, two or more races), missing], and mother’s education (some high school or less, high school diploma, at least some college, bachelor’s degree or more, missing). We also adjusted for residence at birth in a census block group with a majority of non-Hispanic White residents according to the 2000 Census (≤50 %, >50 %) and residence at birth in a census block group where ≥ 23.59 % of the population was below the poverty threshold in the 2000 Census (yes, no), using the same variable definitions as used in the report on indoor perc concentrations in NYC residential buildings with CL-DCs by McDermott et al. (McDermott et al., 2005) To examine a potential non-linear relationship between early-life perc exposure and disease endpoints, we fit 3-knot restricted cubic spline models using data from participants with predicted perc concentrations > 0 μg/m^3^, restricting to disease endpoints with ≥ 20 exposed cases.

We also conducted analyses stratifying the aforementioned dichotomized census block group characteristics by race and ethnicity and poverty level, as well as by mother’s race and ethnicity (non-Hispanic White, non-Hispanic Black, Hispanic, other), mother’s education (high school diploma or less, more than high school diploma), and borough (Manhattan, Bronx, Brooklyn; no perc-exposed cases among participants from Queens or Staten Island). In addition, we conducted analyses subgrouping CL-DC exposure by the year when CL-DCs were built based on MapPLUTO data (no exposure, exposed in CL-DCs built ≤ 1930, exposed in CL-DCs built > 1930; 1930 was the median year of construction among controls), as the age of a building has been linked to an increased potential for fugitive perc releases from CL-DCs. (US Environmental Protection Agency, 2005) We also conducted analyses of cases subgrouped by age at diagnosis (≤5, >5–15, >15 years) using multivariable polytomous regression models. Lastly, we examined the distribution of controls with high perc exposure (estimated perc levels > 44 μg/m^3^; n = 2,267) across socioeconomic factors.

## 3. Results

Selected characteristics of the study controls and two case groups are summarized in Table 1. Compared to controls, cases of childhood cancer were more likely to be male, and their mothers more likely to be non-Hispanic White and to have lived at the time of birth in a census block group with a majority-White population, based on 2000 Census data. Cases of birth defects were more likely than controls to be male and to have been born in the Bronx, and their mothers were more likely to have been aged ≥ 35 years at birth, to be non-Hispanic Black, to have not received a high school diploma at the time of birth, and to have lived in a census block group with a population of majority non-White race and ethnicity or with at least a quarter of residents living in poverty.

As expected, the prevalence of early-life indoor exposure to perc from a CL-DC was low, with 5403 of 591,196 controls (0.9 %) assessed as having lived in a building with a CL-DC at birth. In a comparison of controls with and without early-life exposure to a CL-DC (Supplementary Table S2), we observed that exposed controls were far more likely to have lived in Manhattan. The mothers of exposed controls were more likely to be non-Hispanic White, college-educated, aged ≥ 35 at birth, and to have lived at birth in census block groups that were majority-White and with fewer than a quarter of residents in poverty.

In our case-control analysis of childhood cancers (Table 2) we did not observe an association with early-life residence in buildings with a CL-DC for childhood cancer overall (OR = 0.9, 95 % CI = 0.7, 1.3 in the fully adjusted model) nor for acute lymphoblastic leukemia (OR = 0.9, 95 % CI = 0.5, 1.7), and there was no evidence of an exposure–response relationship across levels of predicted indoor perc concentrations. We observed similar findings in analyses subdividing cases by age at diagnosis for overall cancer (Supplementary Table S3) and ALL (age at diagnosis ≤ 5, OR = 1.2, 95 % CI = 0.6, 2.4; insufficient case numbers to evaluate other age groups).

We also found no association with residence in buildings with a CL-DC in early life for birth defects overall (OR = 1.0, 95 % CI = 1.0, 1.1) and for most of the specific evaluated defects (Fig. 1, Supplementary Table S4, Supplementary Fig. S2). However, exposure was strongly associated with aortic valve stenosis (AVS, OR = 3.1, 95 % CI = 1.6, 5.9, Table 3 and Supplementary Table S4), with a P-value (P = 0.0006) that remained statistically significant after a Bonferroni correction to account for the total number of evaluated endpoints (0.05/26 = 0.0019). We also observed an exposure–response relationship with AVS, with a stronger association for higher predicted indoor perc concentrations (≤44 μg/m^3^, OR = 2.7, 95 % CI = 1.0, 7.4; >44 μg/m^3^, OR = 3.9, 95 % CI = 1.6, 9.5). In restricted cubic spline models, we observed a suggestive positive association with estimated perc levels for cardiovascular birth defects, even after excluding AVS (Supplementary Fig. S2); however, the association with cardiovascular birth defects was null when comparing ever vs. never perc exposure (Supplementary Table S4). We also observed suggestive but imprecise positive associations with birth defects of the eye, other syndromes or malformations, and inguinal hernia in both restricted cubic spline models (Supplementary Fig. S2) and when comparing ever vs. never exposure (Supplementary Table S4).

**Fig. 2.**
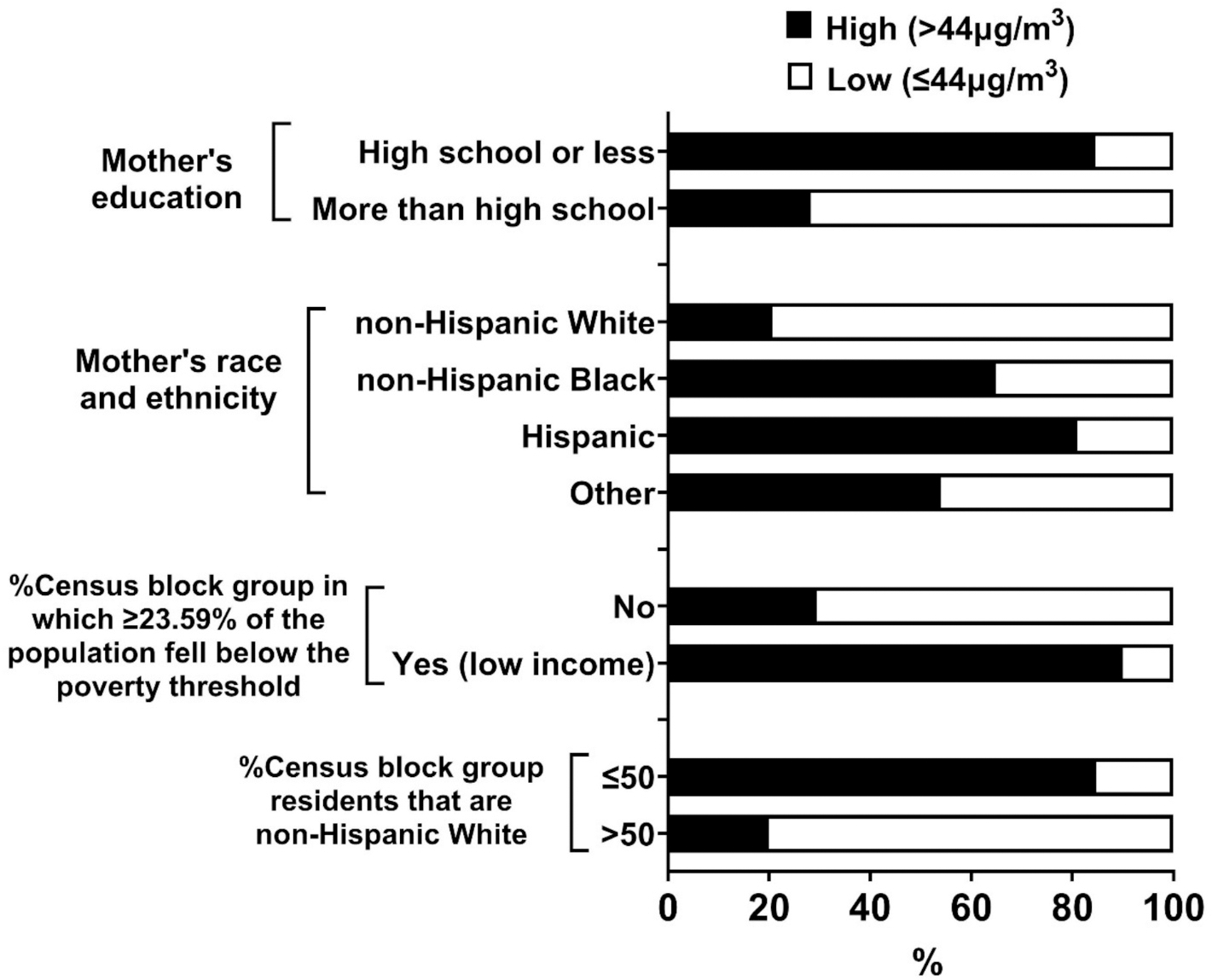
Distribution of controls exposed to high (>44 μg/m^3^) vs. low (≤44 μg/m^3^) by socioeconomic factors. Other in mother’s race and ethnicity includes American Indian and Alaska Native, Native Hawaiian and other Pacific Islander, some other race, and two or more races.

When we further investigated the relationship between early-life residence in CL-DCs and AVS across participant strata (Table 4), we observed evidence of effect modification by socioeconomic factors, with a stronger association observed for participants whose mother was non-Hispanic Black (OR = 9.6, 95 % CI = 3.0, 30.8; non-Hispanic White mother: OR = 2.1, 95 % CI = 0.9, 6.0), who did not attend college (OR = 4.7, 95 % CI = 1.9, 11.5; mother who attended college: OR = 2.4, 95 % CI = 0.9, 6.0), who lived in census block groups that were majority non-White (OR = 4.0, 95 % CI = 1.6, 9.7; majority-White census block group: OR = 2.3, 95 % CI = 0.9, 5.9), and who lived in CL-DCs built in 1930 or earlier (OR = 4.5, 95 % CI = 2.0, 10.1; later than 1930: OR = 2.0, 95 % CI = 0.6, 6.5, Table 3), or to have one-quarter or more of residents in poverty (OR = 4.6, 95 % CI = 1.7, 12.6; <25 % of residents in poverty: OR = 2.4, 95 % CI = 1.0, 5.5). We could not further analyze whether these patterns were similar among highly-exposed groups due to small numbers; however, we observed that among exposed controls, a higher proportion of individuals with these characteristics (i.e., with non-Hispanic Black mothers, mothers who did not attend college, and mothers residing in predominantly non-White and lower income census blocks) had predicted perc exposure levels above the median concentration (Fig. 2).

## 4. Discussion

This study is, to our knowledge, the first to investigate early-life exposure to perc from maternal residence in a building with a CL-DC and risks of childhood cancers and birth defects. We observed a strong association with increased risk of AVS, with evidence of an exposure–response relationship in relation to the predicted indoor concentration of perc. We also observed evidence of effect modification, with stronger associations for AVS observed for participants whose mothers were non-Hispanic Black, had not attended college and/or lived in neighborhoods with higher proportions of majority non-White groups or residents in poverty. We did not observe associations with childhood cancers or other birth defects.

In interpreting these findings, it is informative to consider the study’s strengths and limitations. The population of NYC is well suited to address this research question given its large size and high density, as well as the availability of permitting records regarding perc use in dry cleaning facilities and existing survey data on indoor perc concentrations in residential buildings with CL-DCs. With over 5,000 childhood cancer cases, 170,000 cases with a birth defect and nearly 600,000 controls, the study is one of the largest to investigate early-life perc exposures in relation to these diseases. Despite the large sample size, our analysis was limited by small numbers of exposed cases given the rarity of the exposure. Moreover, we acknowledge the potential for misclassification of birth defect diagnoses given the passive nature of the Birth Defects Registry, and the absence of birth defect ascertainment for non-liveborn outcomes. The use of address information from birth certificates and permitting records for dry-cleaning facilities using perc provided a unique opportunity to identify pre- and perinatal exposure to perc from residence in a building with a CL-DC. However, we acknowledge limitations in that we were unable to utilize direct exposure measurements of indoor air concentrations of perc or other solvents and adjust for factors, such as building characteristics (e.g., renovations) or individual behaviors (e.g., manual ventilation), which might affect exposure levels. This exposure information was particularly well-suited for capturing the relevant etiologic window for birth defects. For childhood cancers, the exposure assessment approach may have been less suitable, to the extent that the window of etiologically relevant exposure extends into later childhood. Additionally, as AVS represents one of 26 endpoints investigated in this study in relation to early-life perc exposure from CL-DCs, we cannot rule out the possibility that the association observed for this birth defect is a chance finding. However, it is notable that the significance level of this association survives a Bonferroni correction accounting for the total number of endpoints included in the analysis and that we observed a stronger association with higher predicted indoor perc concentration.

Epidemiologic findings from two studies of maternal occupational exposures offer support for an association between high levels of chlorinated solvent exposure during pregnancy and AVS. In the Baltimore-Washington Infant Study, a population-based case-control study of congenital heart disease and environmental factors, aortic stenosis risk was associated with maternal exposure to solvents used in degreasing, which includes perc and other chlorinated solvents (OR = 12.5, 95 % CI = 1.6–100). (Loffredo et al., 1991; Ferencz, 1997) Similarly, an investigation within the U.S. multi-center National Birth Defects Prevention Study (2,047 cases and 2,951 controls) found an increased risk of aortic stenosis in relation to occupational exposure to chlorinated solvents (OR = 2.2, 95 % CI = 0.9, 5.7). (Gilboa et al., 2012) Two other U.S. studies that investigated birth defects in relation to high levels of environmental perc releases, in Cape Cod, Massachusetts and Endicott, New York, did not observe associations with overall cardiac defects, although these studies were relatively small in size, each with fewer than 1,500 exposed women who gave birth during the study period, which precluded analyses of associations with uncommon heart defects such as AVS. (Aschengrau et al., 2009; Forand et al., 2012) There is inadequate experimental evidence that perc is a cardiac teratogen, (US Environmental Protection Agency, 2020) although some toxicologic studies of trichloroacetic acid and dichloroacetic acid, metabolites of perc and trichloroethylene, have reported increased defects in cardiac development in rats after high-dose maternal gavage or drinking water exposure. (Makris et al., 2016).

In stratified analyses, we observed stronger associations between potential early-life perc exposure and AVS among individuals whose mothers were non-Hispanic Black, non-college educated and who lived at the time of birth in neighborhoods with higher proportions of low-income or majority non-White residents. These patterns are consistent with the findings from the NYC survey of residential buildings with CL-DCs by McDermott et al., (McDermott et al., 2005) with higher indoor perc concentrations in buildings situated in low-income and majority non-White neighborhoods. Similarly, among study controls with early-life exposure to CL-DCs, individuals with these characteristics were more likely to live at birth in a building with an above-median predicted indoor perc concentration. The NYC building survey was not designed to identify potential building-specific causes of elevated indoor perc concentrations. However, subsequent inspections of affected buildings by NYSDOH and NYCDOHMH found that higher indoor air perc levels were potentially due to poor maintenance and/or faulty operation of dry cleaner vapor barrier enclosures, as well as poor building ventilation and/or maintenance. (Storm et al., 2013) Older buildings may also be more susceptible to elevated perc concentrations, given the potential for poorly sealed pipe chases and more cracks in walls and ceilings, which can allow perc to transport to adjacent residences. (US Environmental Protection Agency, 2005) Residential buildings in majority non-White and low-income neighborhoods often have structural characteristics that can contribute to indoor releases of fugitive perc emissions (e.g., ineffective ventilation and air flow, poor repair, smaller apartments). (Storm et al., 2013) We thus hypothesize that the stronger associations with AVS for strata involving mothers who are non-Hispanic Black, non-college educated and/or living in low-income neighborhoods are due to the potential for higher indoor perc emissions from CL-DCs situated in poorly maintained buildings. Taken together, the fact that strata where we observed stronger associations with AVS are determinants of elevated indoor perc concentrations in residential buildings with CL-DCs offers additional support for the plausibility of our findings.

In conclusion, in this case-control study we observed an association between early-life residential exposure to perc from CL-DCs and AVS risk, particularly for participants with characteristics related to residence in poorer and majority non-White neighborhoods. In view of this finding, additional investigations of high maternal exposures to perc and other chlorinated solvents – - both occupational and environmental – - are needed to clarify this relationship. The use of perc in dry-cleaning facilities situated within residential buildings has been prohibited in the United States since Dec 21, 2020. (US Environmental Protection Agency) To our knowledge, France is the only other country that has enacted similar regulations; (French Minister of Ecology, 2013) it is thus likely that elevated residential exposures to perc from CL-DCs continue to be an issue internationally.

## Supplementary Material

MMC1

Appendix A. Supplementary data

Supplementary data to this article can be found online at https://doi.org/10.1016/j.envint.2025.109600.

## Figures and Tables

**Table 1:** Selected characteristics among controls, overall childhood cancers, and total birth defects.

	Controls (n = 596,599)		Overall childhood cancers (n = 5,334)		Total birth defects (n = 171,553)	
	N	%	N	%	N	%

**Borough**						
Manhattan	101,949	17.1 %	935	17.5 %	27,378	16.0 %
Bronx	110,522	18.5 %	1,001	18.8 %	38,943	22.7 %
Brooklyn	211,517	35.5 %	1,815	34.0 %	62,920	36.7 %
Queens	142,742	23.9 %	1,248	23.4 %	35,142	20.5 %
Staten Island	29,869	5.0 %	335	6.3 %	7,170	4.2 %
**Year of birth**						
<1995	132,359	22.2 %	1,641	30.8 %	39,903	23.3 %
1995-<2002	149,860	25.1 %	1,784	33.4 %	37,742	22.0 %
2002-<2010	160,309	26.9 %	1,295	24.3 %	44,024	25.7 %
2010–2016	154,071	25.8 %	614	11.5 %	49,884	29.1 %
**Mother’s age**						
<25	128,265	21.5 %	1,126	21.1 %	34,576	20.2 %
25–29	161,099	27.0 %	1,434	26.9 %	45,052	26.3 %
30–34	152,051	25.5 %	1,407	26.4 %	44,029	25.7 %
≥35	106,762	17.9 %	939	17.6 %	34,978	20.4 %
Missing	48,422	8.1 %	428	8.0 %	12,918	7.5 %
**Mother’s education**						
Some High School or less	144,513	24.2 %	1,289	24.2 %	45,227	26.4 %
High School Diploma/GED	186,680	31.3 %	1,800	33.7 %	52,553	30.6 %
At least some College	114,421	19.2 %	986	18.5 %	33,228	19.4 %
Bachelor’s Degree or more	140,989	23.6 %	1,183	22.2 %	36,943	21.5 %
Missing	9,996	1.7 %	76	1.4 %	3,602	2.1 %
**Mother’s race and ethnicity**						
Non-Hispanic White	165,390	27.7 %	1,750	32.8 %	42,824	25.0 %
Non-Hispanic Black	152,791	25.6 %	1,183	22.2 %	56,699	33.1 %
Hispanic	197,284	33.1 %	1,823	34.2 %	54,196	31.6 %
Other^a^	79,196	13.3 %	569	10.7 %	17,252	10.1 %
Missing	1,938	0.3 %	9	0.2 %	582	0.3 %
**Infant sex**						
Male	302,608	50.7 %	2,797	52.4 %	100,632	58.7 %
Female	293,991	49.3 %	2,537	47.6 %	70,921	41.3 %
**%Census block group residents that are non-Hispanic White**						
≤25 %	252,307	42.3 %	2,061	38.6 %	82,409	48.0 %
26 %-50 %	132,701	22.2 %	1,179	22.1 %	35,662	20.8 %
51 %-75 %	93,023	15.6 %	865	16.2 %	23,453	13.7 %
76 %-100 %	118,040	19.8 %	1,226	23.0 %	29,863	17.4 %
Missing	528	0.1 %	<16	0.1 %	166	0.1 %
**%Census block group in which ≥ 23.59 %^b^ of the population fell below the poverty threshold**						
No	320,442	53.7 %	2,921	54.8 %	85,672	49.9 %
Yes (low income)	276,157	46.3 %	2,413	45.2 %	85,881	50.1 %

a Other includes non-Hispanic Asian, American Indian and Alaska Native, Native Hawaiian and other Pacific Islander, some other race, and two or more races.

b Cutpoint was selected for consistency with that used in McDermott et al.

**Table 2:** Associations between perc exposure, overall childhood cancer, and acute lymphoblastic leukemia.

	Residence in buildings with CL-DCs			
	Cases N (%)	Controls N (%)	Adjusted OR^a^ (95 % CI)	Adjusted OR^b^ (95 % CI)

**Overall childhood cancer**				
No exposure	5287 (99.1)	591196 (99.1)		
Any	47 (0.9)	5403 (0.9)	1.0 (0.7, 1.3)	0.9 (0.7, 1.3)
*By predicted perc concentration*				
≤44^c^ μg/m^3^	15 (0.3)	2276 (0.4)	0.8 (0.5, 1.3)	0.7 (0.4, 1.2)
>44 μg/m^3^	23 (0.4)	2248 (0.4)	1.1 (0.8, 1.7)	1.1 (0.8, 1.7)
P-trend			0.89	1.00
*By year building built*				
≤1930^c^	27 (0.5)	2276 (0.4)	1.3 (0.9, 1.9)	1.3 (0.9. 1.9)
>1930	12 (0.2)	2311 (0.4)	0.6 (0.4, 1.1)	0.6 (0.3, 1.0)
**Acute lymphoblastic leukemia**				
No exposure	1160 (99.1)	591196 (99.1)		
Any	10 (0.9)	5403 (0.9)	1.0 (0.5, 1.8)	0.9 (0.5, 1.7)
*By predicted perc concentration*				
≤44^c^ μg/m^3^	<10^d^ (0.2)	2276 (0.4)	0.5 (0.1, 1.8)	0.4 (0.1, 1.6)
>44 μg/m^3^	<10 (0.5)	2248 (0.4)	1.4 (0.6, 3.0)	1.3 (0.6, 3.0)
P-trend			0.83	0.95
*By year building built*				
Year built ≤ 1930^c^	<10 (0.4)	2276 (0.4)	1.1 (0.5, 2.7)	1.1 (0.4, 2.6)
Year built > 1930	<10 (0.3)	2311 (0.4)	0.7 (0.2, 2.1)	0.6 (0.2, 1.9)

Abbreviations: co-located dry-cleaning facility, CL-DC; odds ratio, OR; confidence interval, CI.

a Adjusting for year of birth (<1995, 1995-<2002, 2002-<2010, 2010 or later).

b Additionally adjusting for Borough (Manhattan, Bronx, Brooklyn, Queens, Staten Island),, mother’s age (<25, 25–30, 30–34, >35, missing), mother’s race and ethnicity (non-Hispanic White, non-Hispanic Black, Hispanic, other, missing), infant sex (male, female), mother’s education (some high school or less, high school diploma, at least some college, bachelor’s degree or more, missing), %Census block group residents that are non-Hispanic White (≤50, >50), %Census block group in which ≥ 23.59 % of the population fell below the poverty threshold (no, yes).

c Mid-point among controls.

d Numbers less than 10 are suppressed, following the regulations imposed by New York State Department of Health.

**Table 3:** Associations between perc exposure and aortic valve stenosis.

	Residence in buildings with CL-DCs			
	Cases N (%)	Controls N (%)	Adjusted OR^a^ (95 % CI)	Adjusted OR^b^ (95 % CI)

No exposure	361 (97.3)	591196 (99.1)		
Any exposure	10 (2.7)	5403 (0.9)	**3.0 (1.6, 5.7)**	**3.1 (1.6, 5.9)**
*By predicted perc concentration*				
≤44^c^ μg/m^3^	<10^d^ (1.1)	2276 (0.4)	**2.9 (1.1, 7.7)**	**2.7 (1.0, 7.4)**
>44 μg/m^3^	<10 (1.3)	2248 (0.4)	**3.6 (1.5, 8.8)**	**3.9 (1.6, 9.5)**
P-trend			**0.0005**	**0.0004**
*By year building built*				
≤1930^c^	<10 (1.6)	2276 (0.4)	**4.3 (1.9, 9.7)**	**4.5 (2.0. 10.1)**
>1930	<10 (0.8)	2311 (0.4)	2.1 (0.7, 6.6)	2.0 (0.6, 6.5)

Abbreviations: co-located dry-cleaning facility, CL-DC; odds ratio, OR; confidence interval, CI.

d Numbers less than 10 are suppressed, following the regulations imposed by New York State Department of Health.

a Adjusting for year of birth (<1995, 1995-<2002, 2002-<2010, 2010 or later).

b Additionally adjusting for Borough (Manhattan, Bronx, Brooklyn, Queens, Staten Island),, mother’s age (<25, 25–30, 30–34, >35, missing), mother’s race and ethnicity (non-Hispanic White, non-Hispanic Black, Hispanic, other, missing), infant sex (male, female), mother’s education (some high school or less, high school diploma, at least some college, bachelor’s degree or more, missing), %Census block group residents that are non-Hispanic White (≤50, >50), %Census block group in which ≥ 23.59 % of the population fell below the poverty threshold (no, yes).

c Mid-point among controls.

**Table 4:** Associations between perc exposure and aortic valve stenosis, stratified by maternal and neighborhood social factors.

	Residence in buildings with CL-DCs					
	Exposed cases N (% of cases)	Unexposed cases N (% of cases)	Exposed controls N (% of controls)	Unexposed controls N (% of controls)	Adjusted OR^a^ (95 % CI)	Adjusted OR^b^ (95 % CI)

**Stratifying factors**						
**%Census block group residents that are non-Hispanic White**						
≤50	<10^f^ (2.3)	209 (97.7)	2,498 (0.6)	382,627 (99.4)	**3.7 (1.5, 9.0)**	**4.0 (1.6, 9.7)**
>50	<10 (3.2)	151 (96.8)	2,905 (1.4)	208,158 (98.6)	2.4 (0.97, 5.8)	2.3 (0.9, 5.9)
P-interaction					**0.02**	0.05
**%Census block group in which ≥ 23.59 %^c^ of the population fell below the poverty threshold**						
No	<10 (2.7)	214 (97.3)	3,577 (1.1)	316,865 (98.9)	**2.5 (1.1, 5.6)**	**2.4 (1.0, 5.5)**
Yes (low income)	<10 (2.6)	147 (97.4)	1,826 (0.7)	274,331 (99.3)	**4.1 (1.5, 11.0)**	**4.6 (1.7, 12.6)**
P-interaction					**0.01**	**0.007**
**Mother’s race and ethnicity**						
Non-Hispanic White	<10 (2.7)	146 (97.3)	2,308 (1.4)	163,082 (98.6)	1.9 (0.7, 5.2)	2.1 (0.9, 6.0)
Non-Hispanic Black	<10 (3.8)	75 (96.2)	730 (0.5)	152,061 (99.5)	**8.3 (2.6, 26.4)**	**9.6 (3.0, 30.8)**
Hispanic	<10 (1.8)	111 (98.2)	1,652 (0.8)	195,632 (99.2)	2.2 (0.5, 8.7)	2.2 (0.5, 9.1)
Other^d^	<10 (3.4)	28 (96.6)	703 (0.9)	78,493 (99.1)	3.9 (0.5, 29.1)	4.5 (0.6, 34.2)
P-interaction					**0.04**	**0.03**
**Mother’s education**						
High school diploma or less	<10 (2.5)	195 (97.5)	1,980 (0.6)	329,213 (99.4)	**4.3 (1.8, 10.5)**	**4.7 (1.9, 11.5)**
More than high school diploma	<10 (3.2)	151 (96.8)	3,358 (1.3)	252,052 (98.7)	**2.5 (1.0, 6.0)**	2.4 (0.9, 6.0)
P-interaction					**0.05**	0.08
**Borough**^e^						
Manhattan	<10 (7.0)	53 (93.0)	3,475 (3.4)	98,474 (96.6)	2.2 (0.8, 6.0)	1.7 (0.6, 4.8)
Bronx	<10 (2.8)	69 (97.2)	689 (0.6)	109,833 (99.4)	**4.7 (1.2, 19.3)**	**5.0 (1.2, 20.6)**
Brooklyn	<10 (2.8)	139 (97.2)	835 (0.4)	210,682 (99.6)	**7.3 (2.7, 19.7)**	**7.5 (2.7, 20.2)**
P-interaction					**0.03**	**0<.0001**

Abbreviations: co-located dry-cleaning facility, CL-DC; odds ratio, OR; confidence interval, CI.

a Adjusting for year of birth (<1995, 1995-<2002, 2002-<2010, 2010 or later).

b Additionally adjusting for Borough (Manhattan, Bronx, Brooklyn, Queens, Staten Island),, mother’s age (<25, 25–30, 30–34, >35, missing), mother’s race and ethnicity (non-Hispanic White, non-Hispanic Black, Hispanic, other, missing), infant sex (male, female), mother’s education (some high school or less, high school diploma, at least some college, bachelor’s degree or more, missing), %Census block group residents that are non-Hispanic White (≤50, >50), %Census block group in which ≥ 23.59 % of the population fell below the poverty threshold (no, yes).

c Cutpoint was selected for consistency with that used in McDermott et al.

d Other includes non-Hispanic Asian, American Indian and Alaska Native, Native Hawaiian and other Pacific Islander, some other race, and two or more races.

e No perc-exposed cases among participants in Queens or Staten Island.

f Numbers less than 10 are suppressed, following the regulations imposed by New York State Department of Health.

## Data Availability

The data used in this study may be requested from New York State Department of Health by contacting Tabassum Insaf (tabassum.insaf@health.ny.gov) and from the US National Cancer Institute (Mark Purdue, purduem@nih.gov). Requests are subject to a methodologically sound proposal and a data use agreement.
