# Supplementary material for: A case-control study of early-life residential exposure to tetrachloroethylene and risks of childhood cancer and birth defects
Source: Environ Int. Author manuscript; Available in PMC 2025 Jul 8. (PMC12235603; doi:10.1016/j.envint.2025.109600)
Supplement: MMC1 [file NIHMS2090324-supplement-MMC1.docx]

**Supplementary Materials**

| **Supplementary Method 1. The New York State Birth Defects Registry background and ascertainment methods** | **Pages 2-3** |
| --- | --- |
| **Supplementary Method 2. Model development methods to estimate perc concentrations** | **Pages 4-6** |
| **Supplementary Table 1. Predicted concentrations of perc by total number of floors of buildings with co-located dry-cleaning facilities** | **Page 7** |
| **Supplementary Table 2. Selected characteristics among controls, stratified by perc exposure** | **Pages 8-9** |
| **Supplementary Table 3. Associations between perc exposure and all childhood cancer, stratified by maternal and neighborhood social factors** | **Pages 10-11** |
| **Supplementary Table 4. OR and 95% CI of cancer and birth defects associated with perc exposure** | **Pages 12-13** |
| **Supplementary Figure 1. Observed and predicted perc concentrations in relationship with total number of floors using loess regression lines** | **Page 14** |
| **Supplementary Figure 2. Associations between predicted perc concentrations and disease endpoints** | **Page 15** |

**Supplementary Method 1. The New York State Birth Defects Registry background and ascertainment methods**

The New York State Birth Defects Registry (BDR) is a population-based registry that began statewide surveillance in 1983, with hospitals and physicians required to report birth defects diagnosed in liveborn children from birth up to age two. The BDR ascertains cases of structural birth defects, chromosomal anomalies, and persistent metabolic defects primarily through passive surveillance (with enhanced active surveillance for a subset of conditions in certain regions of the state since 1997). From 1983 to 2002, reporting to the BDR, mostly by hospitals, was done via paper-based cards that included patient information, ICD-9 codes, and narrative descriptions of the birth defect(s). Beginning in 2002, the BDR began to implement a method of online electronic reporting, with all facilities utilizing this method by 2006. In 2016, regulation changes were implemented to facilitate reporting of additional birth outcomes, an expansion of provider reporting, and reporting up to age 10 for selected conditions. Additionally, by 2016, all hospitals were reporting ICD-10 codes to the Registry along with a narrative description. To ensure the completeness of reporting, the BDR also incorporates additional data sources including statewide hospital discharge data and, beginning in 2008, electronic clinical laboratory data adding supplemental verification of chromosomal and genetic conditions.

Reported defects must meet certain eligibility criteria and are reviewed by Registry staff before being matched to existing cases (children may be reported from more than one institution from birth to age two or ten). Since 1992, trained Registry staff review all ICD codes and narrative descriptions provided by the hospital and assign more specific codes (based on the British Pediatric Association codes (BPA)) for each case. This ensures that 1) birth defects can be classified and grouped in a consistent manner 2) that cases can be categorized as having isolated or multiple defects 3) any known genetic, chromosomal, or syndromic conditions can be included or excluded as needed depending on the analysis. However, as a passive system without case confirmation through medical record review for all cases, there exists uncertainty in the accuracy and completeness of certain diagnoses. Generally, these diagnoses include those that share ICD-9 or ICD-10 codes, are related to prematurity, naturally resolve in the perinatal period or are related to multi-defect syndromes. Lastly, the BDR routinely matches cases with NYS Birth and Death certificates which allows the birth certificate variables to be used in any reports or analyses and helps to eliminate duplicate cases.

The data included in the present analysis include only birth certificate matched, liveborn cases from the passive surveillance database of the BDR with a major reportable birth defect. Since the timeframe of the analysis includes years for which the BDR relied only on ICD-9 codes, case groups for analysis were constructed using both ICD-9 codes and BPA codes.

**Supplementary Method 2. Model development methods to estimate perc concentrations**

We used published indoor tetrachloroethylene measurements reported in New York City during 2001–2003 (McDermott et al., 2005). The measurements were collected from 65 apartments located in 24 residential dry cleaner buildings. For each sampled building, the following factors were reported: the specific floor sampled, the total number of floors, whether a building had a prior complaint regarding facility emissions, whether the 2000 Census Block Group population was majority non-Hispanic White, whether ≥23.59% of the 2000 Census Block Group population fell below the poverty threshold (if yes, we defined as a low-income community). We observed that all buildings located in the low-income communities were located in majority non-White communities and had a total number of floors ≤7. The total number of floors were highly correlated with the number of floor sampled. We performed ten imputations for measurements below the detection limit of 5 μg/m^3^, assuming lognormality of concentrations with confirmed quantile-quantile plot. Each imputation used low-income (yes, no) as a predictor and building ID to account for measurements from the same building. We conducted mixed-effects linear regression models with log-transformed concentrations as the dependent variable and building ID and floor ID as random effects. We separately tested whether these CL-DCs were located in a majority non-White (yes/no) or low-income community (yes/no), prior complaint filed (yes/no), the number of floor sampled, and total number of floors as fixed effects. We observed that natural log transformed concentrations had a non-linear relationship with the number of floor sampled and total number of floors graphically, where the transformed concentrations decreased in a steep slope and then stabilized. Thus, we conducted three types of transformation for the number of floor sampled and total number of floors: inverse transformation, b-spline analysis (with knots at 25^th^, 50^th^ and 75^th^ percentile), and piecewise linear splines. We applied the models to each of the ten imputed datasets and used testEstimate function in R package mitml to synthesize the overall parameter estimates. The overall Aikake Information Criterion (AIC) was calculated as the average of the AICs of each model. Variables significantly associated with perc concentrations in univariable models were selected as the potential predictors and added to the multi-variable model in order of lowest to the highest model AIC as a main effect. The final model with the lowest AIC was used to predict the concentration (Table A). All analyses above were conducted in R version 4.3.1.

**Table A. Associations between indoor perc concentrations (McDermott et al., 2005) and characteristics of CL-DCs in univariate models**

| **Characteristics of CL-DCs** | **N** | **ß** | **P-value** | **AIC** |
| --- | --- | --- | --- | --- |
| Total number of floors in the building (Floor_total_) |  |  |  |  |
| Intercept |  | 1.00 | 0.09 | 271 |
| $\frac{1}{{Floor}_{total}}$ | 65 | 19.45 | <0.001 |  |
|  |  |  |  |  |
| Number of the floor being sampled (Floor_sampled_) |  |  |  |  |
| Intercept |  | 3.14 | <0.001 | 291 |
| $\frac{1}{{Floor}_{sampled}}$ | 65 | 1.43 | 0.26 |  |
|  |  |  |  |  |
| Building located in low-income community |  |  |  | 281 |
| Intercept |  | 2.91 | <0.001 |  |
| Low income (reference: no) | 11 | 3.15 | <0.001 |  |
|  |  |  |  |  |
| Building located in minority community |  |  |  | 289 |
| Intercept |  | 2.86 | <0.001 |  |
| Minority  (reference: no) | 29 | 1.69 | 0.03 |  |
|  |  |  |  |  |
| Building prior complaint |  |  |  | 293 |
| Intercept |  | 3.78 | <0.001 |  |
| Complaint (reference: no) | 21 | -0.47 | 0.60 |  |

ß, estimated coefficient in the regression model with natural log-transformed concentration as the dependent variable; AIC, aikake information criterion.

**Supplementary Table 1. Predicted concentrations of perc by total number of floors of buildings with co-located dry-cleaning facilities**

| **Total number of floors** | **Predicted concentrations (μg/m^3^)** |
| --- | --- |
| 3 | 1780 |
| 4 | 350 |
| 5 | 130 |
| 6 | 70 |
| 7 | 44 |
| 8 | 31 |
| 9 | 24 |
| 10 | 19 |
| 11 | 16 |
| 12 | 14 |
| 13 | 12 |
| 14 | 11 |
| 15 | 9.9 |
| 16 | 9.2 |
| 17 | 8.5 |
| 18 | 8.0 |
| 19 | 7.6 |
| 20 | 7.2 |
| 21 | 6.9 |
| 22 | 6.6 |
| 23 | 6.3 |
| 24 | 6.1 |
| 25 | 5.9 |
| 26 | 5.7 |

**Supplementary Table 2. Selected characteristics among controls, stratified by maternal residence at birth in a building with a co-located dry-cleaning facility using perc.**

|  | **Residence in co-located dry-cleaning facility using perc** | | | |
| --- | --- | --- | --- | --- |
|  | **No** |  | **Yes** |  |
| **All** | 591,196 |  | 5,403 |  |
|  |  |  |  |  |
| **Borough** |  |  |  |  |
| Manhattan | 98,474 | 16.7% | 3,475 | 64.3% |
| Bronx | 109,833 | 18.6% | 689 | 12.8% |
| Brooklyn | 210,682 | 35.6% | 835 | 15.5% |
| Queens | 142,343 | 24.1% | 399 | 7.4% |
| Staten Island | 29,864 | 5.1% | <10 |  |
|  |  |  |  |  |
| **Year of birth** |  |  |  |  |
| <1995 | 131,216 | 22.2% | 1,143 | 21.2% |
| 1995-<2002 | 148,532 | 25.1% | 1,328 | 24.6% |
| 2002-<2010 | 158,876 | 26.9% | 1,433 | 26.5% |
| 2010-2016 | 152,572 | 25.8% | 1,499 | 27.7% |
|  |  |  |  |  |
| **Mother's age** |  |  |  |  |
| <25 | 175,486 | 29.7% | 1,060 | 19.6% |
| 25-29 | 159,906 | 27.0% | 1,193 | 22.1% |
| 30-34 | 150,298 | 25.4% | 1,753 | 32.4% |
| 35 & over | 105,366 | 17.8% | 1,396 | 25.8% |
| Missing | 140 | 0.0% | <10 |  |
|  |  |  |  |  |
| **Mother's education** |  |  |  |  |
| Some High School or less | 143,556 | 24.3% | 957 | 17.7% |
| High School Diploma/GED | 185,657 | 31.4% | 1,023 | 18.9% |
| At least some College | 113,689 | 19.2% | 732 | 13.5% |
| Bachelor's Degree or more | 138,363 | 23.4% | 2,626 | 48.6% |
| Missing | 9,931 | 1.7% | 65 | 1.2% |
|  |  |  |  |  |
| **Mother's race and ethnicity** |  |  |  |  |
| Non-Hispanic White | 163,082 | 27.6% | 2,308 | 42.7% |
| Non-Hispanic Black | 152,061 | 25.7% | 730 | 12.5% |
| Hispanic | 195,632 | 33.1% | 1,652 | 30.6% |
| Other^a^ | 78,493 | 13.3% | 703 | 13.0% |
| Missing | 1,928 | 0.3% | 10 | 0.2% |
|  |  |  |  |  |
| **Infant sex** |  |  |  |  |
| Male | 299,884 | 50.7% | 2,724 | 50.4% |
| Female | 291,312 | 49.3% | 2,679 | 49.6% |
|  |  |  |  |  |
| **%Census block group residents that are non-Hispanic White** |  |  |  |  |
| ≤25% | 250,592 | 42.4% | 1,715 | 31.7% |
| 26%-50% | 131,918 | 22.3% | 783 | 14.5% |
| 51%-75% | 92,092 | 15.6% | 931 | 17.2% |
| 76%-100% | 116,066 | 19.6% | 1,974 | 36.5% |
| Missing | 528 | 0.1% | 0 |  |
|  |  |  |  |  |
| **%Census block group in which ≥23.59%^b^ of the population fell below the poverty threshold** |  |  |  |  |
| No | 316,865 | 53.6% | 3,577 | 66.2% |
| Yes (low income) | 274,331 | 46.4% | 1,826 | 33.8% |

^a^Other includes non-Hispanic Asian, American Indian and Alaska Native, Native Hawaiian and other Pacific Islander, some other race, and two or more races.

^b^Cutpoint was selected for consistency with that used in McDermott et al.

**Supplementary Table 3. Associations between perc exposure and all childhood cancer, stratified by maternal and neighborhood social factors**

|  | **Residence in buildings with CL-DCs** | | | |  |  |
| --- | --- | --- | --- | --- | --- | --- |
|  | **Exposed cases**  **N (% of cases)** | **Unexposed cases**  **N (% of cases)** | **Exposed controls**  **N (% of controls)** | **Unexposed controls**  **N (% of controls)** | **Adjusted OR^a^**  **(95% CI)** | **Adjusted OR^b^**  **(95% CI)** |
| **Stratifying factors** |  |  |  |  |  |  |
| **%Census block group residents that are non-Hispanic White** |  |  |  |  |  |  |
| ≤50 | 32 (1.0) | 3,210 (99.0) | 2,498 (0.6) | 382,627 (99.4) | **1.5 (1.1, 2.1)** | **1.5 (1.0, 2.1)** |
| >50 | 15 (0.7) | 2,076 (99.3) | 2,905 (1.4) | 208,158 (98.6) | **0.5 (0.3, 0.9)** | **0.5 (0.3, 0.9)** |
| P-interaction |  |  |  |  | **0.06** | **0.02** |
|  |  |  |  |  |  |  |
| **%Census block group in which ≥23.59% of the population fell below the poverty threshold** |  |  |  |  |  |  |
| No | 24 (0.8) | 2,897 (99.2) | 3,577 (1.1) | 316,865 (98.9) | 0.8 (0.5, 1.1) | 0.7 (0.5, 1.1) |
| Yes (low income) | 23 (1.0) | 2,390 (99.0) | 1,826 (0.7) | 274,331 (99.3) | 1.4 (0.95, 2.2) | 1.4 (0.9, 2.1) |
| P-interaction |  |  |  |  | 0.13 | 0.1 |
|  |  |  |  |  |  |  |
| **Mother's race and ethnicity** |  |  |  |  |  |  |
| Non-Hispanic White | 15 (0.9) | 1,735 (99.1) | 2,308 (1.4) | 163,082 (98.6) | 0.6 (0.4, 1.1) | 0.6 (0.4, 1.1) |
| Non-Hispanic Black | 10 (0.8) | 1,173 (99.2) | 730 (0.5) | 152,061 (99.5) | **1.8 (0.9, 3.3)** | **1.8 (1.0, 3.4)** |
| Hispanic | 18 (1.0) | 1,805 (99.0) | 1,652 (0.8) | 195,632 (99.2) | 1.2 (0.7, 1.8) | 1.1 (0.7, 1.8) |
| Other^a^ | <10^e^ (0.5) | 566 (99.5) | 703 (0.9) | 78,493 (99.1) | 0.6 (0.2, 1.9) | 0.6 (0.2, 1.9) |
| P-interaction |  |  |  |  | 0.42 | 0.30 |
|  |  |  |  |  |  |  |
| **Mother's education** |  |  |  |  |  |  |
| High school diploma or less | 21 (0.7) | 3,068 (99.3) | 1,980 (0.6) | 329,213 (99.4) | 1.1 (0.7, 1.7) | 1.1 (0.7, 1.7) |
| More than high school diploma | 26 (1.2) | 2,143 (98.8) | 3,358 (1.3) | 252,052 (98.7) | 0.9 (0.6, 1.3) | 0.9 (0.6, 1.3) |
| P-interaction |  |  |  |  | 0.70 | 0.38 |
|  |  |  |  |  |  |  |
| **Child’s age at diagnosis^f^** |  |  |  |  |  |  |
| ≤ 5 years | 20 (0.9) | 2,209 (99.1) | ^-^ | - | 1.0 (0.6, 1.5) | 0.9 (0.6, 1.4) |
| >5-15 years | 19 (0.9) | 2,109 (99.1) | - | - | 1.0 (0.6, 1.6) | 1.0 (0.6, 1.6) |
| >15 years | <10 (0.8) | 969 (99.2) | - | - | 0.9 (0.5, 1.9) | 0.8 (0.4, 1.7) |
| **Borough^d^** |  |  |  |  |  |  |
| Manhattan | 26 (2.8) | 909 (97.2) | 3,475 (3.4) | 98,474 (96.6) | 0.8 (0.6, 1.2) | 0.8 (0.5, 1.2) |
| Bronx | 10 (1.0) | 991 (99.0) | 689 (0.6) | 109,833 (99.4) | 1.6 (0.9, 2.9) | 1.5 (0.8, 2.8) |
| Brooklyn | <10 (0.3) | 1,809 (99.7) | 835 (0.4) | 210,682 (99.6) | 0.8 (0.4, 1.9) | 0.9 (0.4, 2.0) |
| Queens | <10 (0.4) | 1,243 (99.6) | 399 (0.3) | 142,343 (99.7) | 1.5 (0.6, 3.5) | 1.5 (0.6, 3.6) |
| P-interaction |  |  |  |  | 0.40 | 0.77 |

Abbreviations: co-located dry-cleaning facility, CL-DC; odds ratio, OR; confidence interval, CI

^a^Adjusting for year of birth (<1995, 1995-<2002, 2002-<2010, 2010 or later)

^b^Additionally adjusting for Borough (Manhattan, Bronx, Brooklyn, Queens, Staten Island), mother’s age (<25, 25-30, 30-34, >35, missing), mother’s race and ethnicity (non-Hispanic White, non-Hispanic Black, Hispanic, other, missing), infant sex (male, female), mother’s education (some high school or less, high school diploma, at least some college, bachelor’s degree or more, missing), %Census block group residents that are non-Hispanic White (≤50, >50), %Census block group in which ≥23.59% of the population fell below the poverty threshold (no, yes)

^c^Other includes American Indian and Alaska Native, Asian, Native Hawaiian and other Pacific Islander, some other race, and two or more races

^d^No perc-exposed cases among participants in Staten Island.

^e^Numbers less than 10 are suppressed, following the regulations imposed by New York State Department of Health.

^f^Polytomous regression analyses (cases diagnosed ≤5, >5-15, >15 years vs. controls) were conducted.

**Supplementary Table 4. OR and 95% CI of cancer and birth defects^a^ associated with perc exposure**

|  |  |  | **Adjusted^b^** | | | **Adjusted^c^** | | |
| --- | --- | --- | --- | --- | --- | --- | --- | --- |
|  | **Cases**  **N (N_exposed_, %)** | **Controls**  **N (N_exposed_, %)** | **OR** | **95% CI** | | **OR** | **95% CI** | |
| **All cancer** | 5,334 (47, 0.9%) | 596,599 (5,403, 0.9%) | 0.99 | 0.74 | 1.32 | 0.94 | 0.71 | 1.26 |
| Acute lymphoblastic leukemia | 1,170 (10, 0.9%) | 596,599 (5,403, 0.9%) | 0.95 | 0.51 | 1.77 | 0.89 | 0.47 | 1.67 |
| **All birth defects** | 171,553 (1497, 0.9%) | 596,599 (5,403, 0.9%) | 0.96 | 0.91 | 1.02 | 1.01 | 0.95 | 1.07 |
| **Central Nervous System Defects** | 12,641 (98, 0.8%) | 596,599 (5,403, 0.9%) | 0.85 | 0.69 | 1.03 | 0.95 | 0.78 | 1.16 |
| Spina Bifida without Anencephaly | 798 (<10, 0.8%) | 596,599 (5,403, 0.9%) | 0.84 | 0.38 | 1.87 | 1.06 | 0.47 | 2.38 |
| **Orofacial Birth Defects** | 4,021 (33, 0.8%) | 596,599 (5,403, 0.9%) | 0.90 | 0.64 | 1.27 | 0.93 | 0.66 | 1.31 |
| Cleft Lip with Cleft Palate | 1,349 (11, 0.8%) | 596,599 (5,403, 0.9%) | 0.90 | 0.50 | 1.63 | 1.01 | 0.56 | 1.84 |
| Cleft Palate Alone | 1,536 (10, 0.7%) | 596,599 (5,403, 0.9%) | 0.72 | 0.39 | 1.34 | 0.72 | 0.39 | 1.35 |
| **Birth Defects of the Eye** | 2,160 (20, 0.9%) | 596,599 (5,403, 0.9%) | 1.02 | 0.66 | 1.59 | 1.18 | 0.76 | 1.84 |
| Congenital Cataracts | 582 (<10, 1.4%) | 596,599 (5,403, 0.9%) | 1.51 | 0.75 | 3.04 | 1.66 | 0.82 | 3.37 |
| **Cardiovascular Birth Defects** | 48,925 (436, 0.9%) | 596,599 (5,403, 0.9%) | 0.97 | 0.88 | 1.07 | 1.00 | 0.91 | 1.11 |
| Aortic valve stenosis | 371 (10, 2.7%) | 596,599 (5,403, 0.9%) | 3.03 | 1.61 | 5.68 | 3.09 | 1.62 | 5.90 |
| Atrioventricular Septal Defect | 2,541 (19, 0.7%) | 596,599 (5,403, 0.9%) | 0.84 | 0.53 | 1.32 | 0.81 | 0.51 | 1.27 |
| Coarctation of Aorta | 1,490 (19, 1.3%) | 596,599 (5,403, 0.9%) | 1.40 | 0.89 | 2.20 | 1.39 | 0.88 | 2.20 |
| Hypoplastic Left Heart Syndrome | 668 (<10, 0.9%) | 596,599 (5,403, 0.9%) | 0.99 | 0.44 | 2.21 | 1.22 | 0.54 | 2.75 |
| Tetralogy of Fallot | 1,706 (17, 1.0%) | 596,599 (5,403, 0.9%) | 1.10 | 0.68 | 1.78 | 1.14 | 0.71 | 1.85 |
| Pulmonary Valve Atresia and Stenosis | 2,885 (21, 0.7%) | 596,599 (5,403, 0.9%) | 0.80 | 0.52 | 1.23 | 0.82 | 0.53 | 1.27 |
| **Gastrointestinal Birth Defects** | 10,705 (80, 0.7%) | 596,599 (5,403, 0.9%) | 0.82 | 0.66 | 1.03 | 0.90 | 0.72 | 1.13 |
| Biliary Atresia | 436 (<10, 1.6%) | 596,599 (5,403, 0.9%) | 1.77 | 0.84 | 3.74 | 2.07 | 0.97 | 4.40 |
| Esophageal Atresia and Tracheoesophageal Fistula | 739 (<10, 0.9%) | 596,599 (5,403, 0.9%) | 1.05 | 0.50 | 2.21 | 0.93 | 0.44 | 1.97 |
| Small Intestinal Atresia and Stenosis | 1,243 (<10, 0.7%) | 596,599 (5,403, 0.9%) | 0.80 | 0.41 | 1.54 | 0.98 | 0.50 | 1.89 |
| Rectal and Large Intestinal Atresia and Stenosis | 1,285 (<10, 0.5%) | 596,599 (5,403, 0.9%) | 0.51 | 0.23 | 1.15 | 0.58 | 0.26 | 1.30 |
| **Genitourinary Birth Defects** | 35,592 (348, 1.0%) | 596,599 (5,403, 0.9%) | 1.06 | 0.95 | 1.19 | 1.03 | 0.92 | 1.16 |
| Hypospadias - All Degrees (male only) | 9,103 (94, 1.0%) | 596,599 (5,403, 0.9%) | 1.13 | 0.92 | 1.39 | 1.05 | 0.85 | 1.30 |
| Renal Agenesis | 1,160 (12, 1.0%) | 596,599 (5,403, 0.9%) | 1.13 | 0.64 | 2.00 | 1.14 | 0.64 | 2.03 |
| **Musculoskeletal Birth Defects** | 37,579 (360, 1.0%) | 596,599 (5,403, 0.9%) | 1.06 | 0.95 | 1.18 | 1.11 | 0.99 | 1.23 |
| Clubfoot | 4,239 (40, 0.9%) | 596,599 (5,403, 0.9%) | 1.04 | 0.76 | 1.42 | 1.13 | 0.83 | 1.55 |
| Limb Reduction Defects | 1,010 (10, 1.0%) | 596,599 (5,403, 0.9%) | 1.10 | 0.59 | 2.06 | 1.13 | 0.60 | 2.12 |
| Craniosynostosis | 1,638 (16, 1.0%) | 596,599 (5,403, 0.9%) | 1.07 | 0.65 | 1.76 | 1.03 | 0.63 | 1.70 |
| Diaphragmatic Hernia | 644 (<10, 1.1%) | 596,599 (5,403, 0.9%) | 1.20 | 0.57 | 2.53 | 1.30 | 0.61 | 2.77 |
| Inguinal Hernia (female only) | 1,249 (25, 1.0%) | 596,599 (5,403, 0.9%) | 1.22 | 0.72 | 2.07 | 1.48 | 0.87 | 2.53 |
| **Other Syndromes or Malformations** | 2,491 (23, 1.1%) | 596,599 (5,403, 0.9%) | 1.12 | 0.75 | 1.66 | 1.25 | 0.84 | 1.86 |
| **Soft Tissue Conditions** | 2,114 (23, 1.1%) | 596,599 (5,403, 0.9%) | 1.18 | 0.78 | 1.78 | 1.05 | 0.69 | 1.59 |
| **Others (no category)** |  |  |  |  |  |  |  |  |
| Cystic Hygroma | 367 (<10, 1.4%) | 596,599 (5,403, 0.9%) | 1.51 | 0.62 | 3.65 | 1.42 | 0.58 | 3.48 |
| Hemangioma- Major | 1,019 (10, 1.0%) | 596,599 (5,403, 0.9%) | 1.04 | 0.56 | 1.95 | 0.88 | 0.47 | 1.66 |

Abbreviations: odds ratio, OR; confidence interval, CI

^a^We conducted analyses for endpoints with a minimum of five cases with a co-located dry cleaning facility in their residential building at birth

^b^Adjusting for year of birth (<1995, 1995-<2002, 2002-<2010, 2010 or later)

^c^Additionally adjusting for Borough (Manhattan, Bronx, Brooklyn, Queens, Staten Island), , mother’s age (<25, 25-30, 30-34, >35, missing), mother’s race and ethnicity (non-Hispanic White, non-Hispanic Black, Hispanic, other, missing), infant sex (male, female), mother’s education (some high school or less, high school diploma, at least some college, bachelor’s degree or more, missing), %Census block group residents that are non-Hispanic White (≤50, >50), %Census block group in which ≥23.59% of the population fell below the poverty threshold (no, yes)

^d^Numbers less than 10 are suppressed, following the regulations imposed by New York State Department of Health.

**Supplementary Figure 1. Observed^a^ and predicted perc concentrations in relationship with total number of floors using loess regression lines**

**
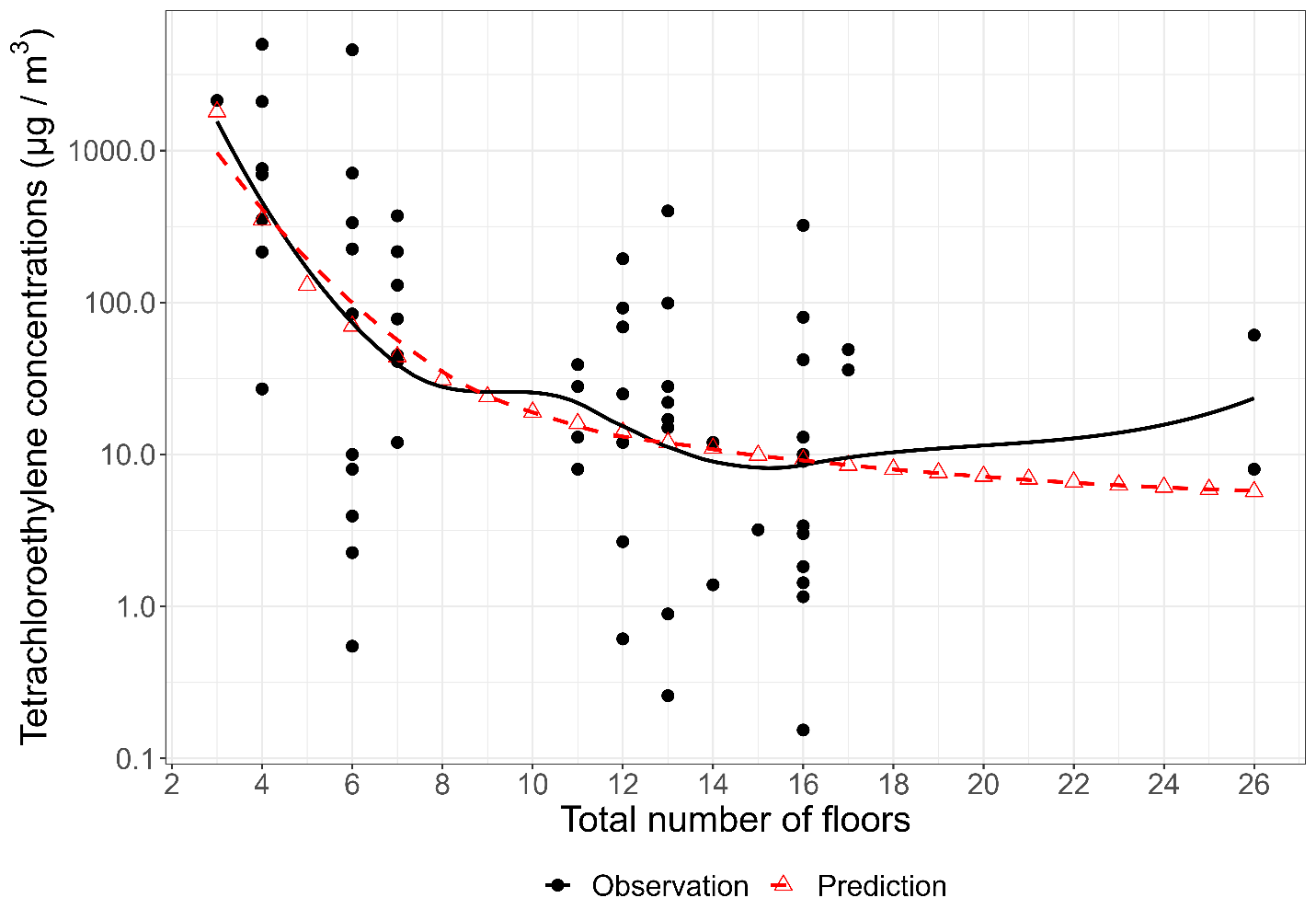
**

^a^Observed data is from the first imputed dataset

**Supplementary Figure 2. Associations^a^ between predicted perc concentrations and disease endpoints. X-axis represents predicted perc concentrations (0-1000 μg/m^3^) and Y-axis represents odds ratio of each disease endpoint.**


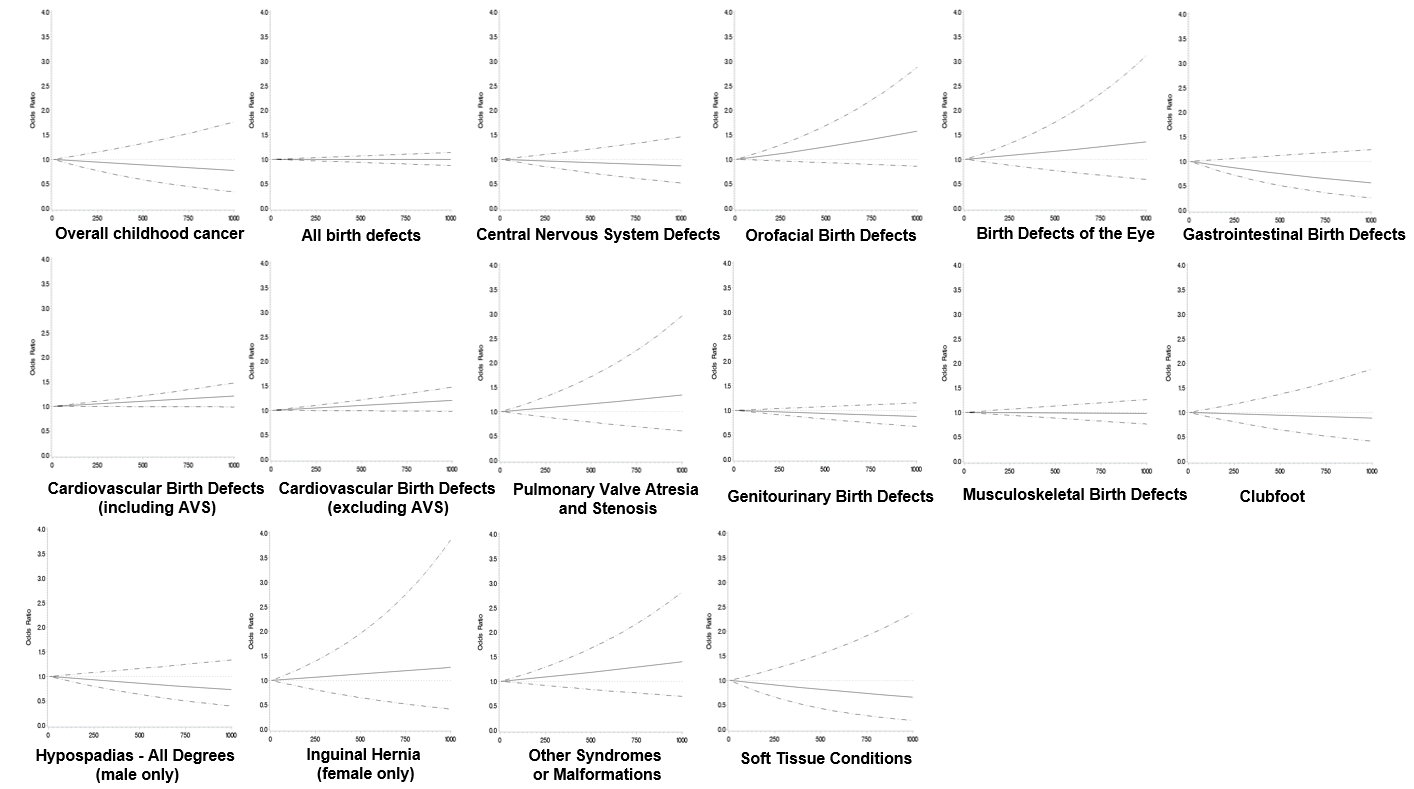


^a^Results are from 3-knot restricted cubic spline models using data from participants with predicted perc concentrations greater than zero, restricting to disease endpoints with ≥20 exposed cases, adjusting for year of birth (<1995, 1995-<2002, 2002-<2010, 2010 or later), Borough (Manhattan, Bronx, Brooklyn, Queens, Staten Island), , mother’s age (<25, 25-30, 30-34, >35, missing), mother’s race and ethnicity (non-Hispanic White, non-Hispanic Black, Hispanic, other, missing), infant sex (male, female), mother’s education (some high school or less, high school diploma, at least some college, bachelor’s degree or more, missing), %Census block group residents that are non-Hispanic White (≤50, >50), %Census block group in which ≥23.59% of the population fell below the poverty threshold (no, yes). The results for inguinal hernia are derived from an unadjusted model due to the presence of zero cells among perc-exposed groups in several covariate categories.
